# Smoking and vaping patterns during pregnancy and the postpartum: A longitudinal UK cohort survey

**DOI:** 10.1016/j.addbeh.2021.107050

**Published:** 2021-12

**Authors:** Katharine Bowker, Sarah Lewis, Michael Ussher, Felix Naughton, Lucy Phillips, Tim Coleman, Sophie Orton, Hayden McRobbie, Linda Bauld, Sue Cooper

**Affiliations:** a Division of Primary Care Research and UK Centre for Tobacco and Alcohol Studies, School of Medicine, University of Nottingham, Nottingham NG7 2RD, UK; b Division of Epidemiology and Public Health and UK Centre for Tobacco and Alcohol Studies, University of Nottingham, Clinical Sciences Building 2, Nottingham City Hospital Hucknall Road, Nottingham NG5 1PB, UK; c Population Health Research Institute, St George's, University of London, London SW17 0RE, UK; d Institute for Social Marketing and Health, University of Stirling, Stirling FK9 4LA, UK; e University of East Anglia, Faculty of Medicine and Health Sciences, Edith Cavell Building, Norwich NR4 7TJ, UK; f National Drug and Alcohol Research Centre, University of New South Wales, Sydney, NSW 2031, Australia; g Usher Institute, College of Medicine and Veterinary Medicine, University of Edinburgh, EH16 4UX, UK

**Keywords:** Pregnancy, Smoking, Vaping, e-Cigarettes, Prevalence, Longitudinal

## Abstract

**Introduction:**

There is limited information about longitudinal patterns of vaping during pregnancy and the postpartum. We describe the prevalence, frequency, and reasons for vaping throughout pregnancy and postpartum. We also describe temporal patterns in pregnant women’s vaping.

**Methods:**

A longitudinal cohort study across England and Scotland, with questionnaires in early pregnancy (8–24 weeks gestation), late pregnancy (34–38 weeks) and 3 months postpartum. A total of 750 women, aged 16 years or over, who were either current smokers, vapers or had smoked in the 3 months before pregnancy, were recruited between June and November 2017.

**Results:**

Vaping prevalence was 15.9% (n = 119/750) in early pregnancy: 12.4% (n = 93/750) were dual users and 3.5% (n = 26/750) exclusive vapers. Late pregnancy vaping prevalence was 17.8% (n = 68/383): 12.5% (n = 48/383) were dual users and 5.2% (n = 20/383) exclusive vapers. Postpartum vaping prevalence was 23.1% (n = 95/411): 14.6% (n = 60/411) were dual users and 8.5% (n = 35/411) exclusive vapers. The most frequently reported reason to vape among all vapers was to quit smoking. A total of 316 women completed all three surveys: 2.6% (n = 8/316) were exclusive vapers in early pregnancy with most remaining exclusive vapers postpartum (n = 6/8, 75%). Of the 11.5% (n = 35/316) dual users in early pregnancy, 31.4% (n = 11/35) were exclusive smokers by the postpartum.

**Conclusion:**

Vaping prevalence was between 15.9% and 23.1% during pregnancy and the postpartum period, and the majority were dual users. Vaping habits of exclusive vapers remains stable throughout pregnancy and the postpartum. However, the vaping habits of dual users varies, with a third exclusively smoking in the postpartum.

## Highlights

• Between 16% and 23% of pregnant smokers and ex-smokers vape during pregnancy.
• Most pregnant vapers also continue to smoke (dual use).
• Vaping habits of exclusive vapers is stable during pregnancy and the postpartum.
• A third of dual users in early pregnancy are exclusively smoking in the postpartum.

## 1 Background

Smoking in pregnancy has adverse health consequences for the woman and baby (Clifford et al., 2012, Cnattingius, 2004, Delpisheh et al., 2007, Gluckman et al., 2008); efforts to eliminate smoking is a public health priority. In England, 10.4% of women self-report smoking at delivery (NHS Digital, 2019) and rates are higher among younger and more deprived women (Health and Social Care Information Centre, 2015, McAndrew et al., 2012). Up to half of women report quitting smoking either just before or around the time of finding out they are pregnant (Orton et al., 2014, Pickett et al., 2003); however, up to 60% of these may relapse in the postpartum(Colman and Joyce, 2003, Cooper et al., 2017, Jones et al., 2016). Exposure to second-hand smoke from postpartum smoking will increase the infant’s risk of sudden infant death, respiratory and ear infections, and asthma (Pugmire, Sweeting, & Moore, 2017). In addition, children of women who smoke cigarettes are more likely to initiate smoking themselves (Leonardi-Bee, Jere, & Britton, 2011).

Electronic cigarette (e-cigarette/vaping) prevalence in England in 2019 was between 5 and 7% for non-pregnant adults (Ann McNeill, Brose, Calder, Bauld, & Robson, 2020). Vaping appears to be an effective aid to assist non-pregnant smokers to quit smoking (Hajek et al., 2019, Hartmann-Boyce et al., 2020). Although not risk free, e-cigarettes, unlike cigarettes, do not release products of combustion (A McNeill et al., 2015). Compared to smoking, vaping exposes non-pregnant adults to lower levels of carcinogens and toxins (Caponnetto et al., 2018, Shahab et al., 2017). Vapers who quit smoking (exclusive vapers) have lower toxicant exposure compared to dual users (those who smoke and vape) (Goniewicz et al., 2018). Exposure to second-hand e-cigarette vapour may also pose less risk than exposure to second-hand cigarette smoke (Hess, Lachireddy, & Capon, 2016). The Royal College of Physicians concluded vaping is unlikely to exceed 5% of the harm from smoking (Royal College of Physicians, 2016). There are limited data on the safety of vaping during pregnancy on the woman or baby (Cardenas et al., 2019, Froggatt et al., 2020, Gillen and Saltzman, 2014, McDonnell et al., 2020). However, it is unlikely that findings regarding vaping safety among non-pregnant populations would be different from pregnant women. There is currently no evidence about the effectiveness of vaping for helping women to stop smoking during pregnancy. Current advice for clinicians caring for pregnant women in the UK supports vaping in order to avoid smoking (Smoking in Pregnancy Challenge Group, 2019).

Cross sectional data on vaping during pregnancy show that prevalence is between 0.6 and 15% (Bowker et al., 2021, Kapaya et al., 2019, Kurti et al., 2017, Liu et al., 2019, Mark et al., 2015, Obisesan et al., 2020, Rollins et al., 2020), and that most pregnant vapers also smoke (dual use) (Bowker et al., 2021, Kapaya et al., 2019, Liu et al., 2019). Such variation in prevalence figures may be influenced by different methods of data collection, recall periods, whether women were asked about use before or at differing timepoints during pregnancy, and variation between countries. There is limited understanding about longitudinal patterns of vaping throughout pregnancy. If e-cigarettes are shown to be less harmful in pregnancy than smoking, they could be a useful tool to help women who cannot quit smoking completely using traditional methods. Finding out why and when pregnant women vape and how this relates to smoking status would help us to understand the context around vaping during pregnancy.

In this longitudinal cohort study, we describe the prevalence, frequency and reasons for vaping throughout pregnancy and the postpartum. We also describe temporal patterns in individuals’ smoking and vaping during pregnancy and postpartum. We describe whether exposure remains stable or varies and how this relates to smoking status. Understanding why women are vaping could help us understand women’s perceptions about the role of e-cigarettes for smoking cessation and whether views vary throughout pregnancy and the postpartum.

## 2 Methods

### 2.1 Study design

A longitudinal cohort study was undertaken; eligible women were 16 years old or over (no upper age limit), 8–24 weeks pregnant and either recent ex-smokers (smoked during the 3 months immediately prior to finding out they were pregnant), current smokers (every day or occasionally) and/or vapers (every day or occasionally). Surveys were conducted in early pregnancy (8–24 weeks gestation) (baseline), late pregnancy (34–38 weeks gestation) and postpartum (3 months postpartum). Women who were unable to read or understand the questionnaires in English or were enrolled in other smoking cessation studies were excluded. A detailed description of the methods and characteristics of the participants recruited is published elsewhere (Bowker et al., 2021). Ethical approval was given by the South West Frenchay Research Ethics Committee. We used “Strengthening the Reporting of Observational Studies in Epidemiology” (STROBE) (von Elm et al., 2007) and “Transparent Reporting of Evaluations with Nonrandomized Designs” (TREND) guidance (Des Jarlais, Lyles, Crepaz, & Group, 2004) to aid the reporting of this study.

### 2.2 Study setting and regimen

Women were recruited between June and November 2017 while attending National Health Service (NHS) hospital antenatal clinics at a range of locations in England and Scotland. Posters were visible in the antenatal clinics and research midwives/nurses promoted the study by handing a questionnaire to women attending clinics. Women completed a screening survey asking about their vaping and smoking status; those eligible and willing then completed a full baseline survey at the same time point (consent was implied through their completion of the questionnaire). They were then asked to give consent to join the longitudinal cohort and be sent follow-up surveys by post or email web-link. Written consent for longitudinal follow-up was taken face-to-face after completing the baseline (early pregnancy) survey; however, if women required more time, they were followed up by telephone, and verbal consent was taken. At each follow-up, participants were sent a prompt by Short Message Service (SMS) texts to enhance response rates, plus one reminder by post, text and/or email. If women failed to respond they were called to complete questions by telephone. Women were offered a £10 high street shopping voucher for completing each survey.

### 2.3 Description of the surveys

The early pregnancy survey included questions on age, gestation, educational attainment, age left education, ethnicity, previous pregnancies and whether pregnancy was planned. All three surveys contained a section about the participant’s experience of using e-cigarettes, smoking behaviour and beliefs. Responses included yes/no answers, Likert scales and multiple-choice options. The two follow-up surveys asked questions about infant feeding methods and the postpartum survey asked about birthweight.

All three surveys asked current vapers about their main reason for vaping, offering eight options. Due to low use of some of the response options, we report the top three responses: to quit smoking, to cut down smoking, to avoid returning to smoking. This latter option could imply women perceived themselves as established ex-smokers or may have been ex-smokers when they started vaping. Our ‘other’ category amalgamates the remaining responses: curiosity, enjoyment, to use when I am not allowed to smoke, don’t know and other (unknown). Women in the postpartum were also given the option ‘to use around my baby’.

Cigarette dependence was assessed using the Heaviness of Smoking Index (HSI) (Heatherton et al., 1991, Heatherton et al., 1989, Riaz et al., 2016) (time to first smoking in the morning and number of cigarettes per day). Cigarettes smoked per day (CPD) were categorised as either “0–10” or “≥11” to distinguish between heavy and light smokers (Husten, 2009); we included zero as some women smoked occasionally but not every day.

The surveys are available online as supplementary information.

### 2.4 Measurements

#### 2.4.1 Smoking and vaping status at baseline

In early pregnancy, vaping status was determined on responses to the following statement: ‘*what best describes your use of e-cigarettes right now?’*. Participants could select one of the following: 1) *I have never heard of e-cigarettes and have never tried them; 2) I have heard of e-cigarettes but have never tried them; 3) I have tried e-cigarettes, but do not use them now; 4) I have tried e-cigarettes and still use them, but not every day; 5) I have tried e-cigarettes and still use them every day.*

Smoking status was based on responses to the following statement: ‘*what best describes your smoking right now?’*. Participants could select one of the following: 1) *I have never smoked;* 2) *I completely stopped smoking more than 3 months before finding out I was pregnant; 3) I completely stopped smoking at some time in the 3 months before finding out I was pregnant;* 4) *I completely stopped smoking after I found out I was pregnant*; *5)I smoke occasionally, but not every day now I am pregnant;* 6) *I smoke every day, but have cut down during my pregnancy;* 7) *I smoke every day, about the same as before my pregnancy;* 8) *I smoke every day, and tend to smoke more than before my pregnancy.*

Ex-smokers were those who reported they were not smoking currently but had done so during the 3 months before finding out they were pregnant. Women who reported vaping daily or occasionally (vape, but not every day) were defined as ‘vapers’. Women who reported that they smoked either daily or occasionally and did not vape (in any capacity), were defined as a ‘smoker’. Smokers who reported that they also vaped (in any capacity) were defined as ‘dual users’. Women who reported that they did not smoke but vaped (in any capacity) were defined as ‘exclusive vapers’.

#### 2.4.2 Smoking and vaping status at follow up

On the follow-up surveys, women were asked ‘*How often do you use an e-cigarette or vaping device now?*’ and could select the following options: 1) *Not used at all; 2) only used once or twice; 3) used occasionally, but less than weekly; 4) used less than daily, but at least once a week; 5) used every day.*

Smoking status was determined on responses to the following statement: ‘*what best describes your smoking right now?’.* Participants could select the following: 1) *I don’t smoke at all; 2) I smoke occasionally, but not every day; 3) I smoke every day, but have cut down during my pregnancy; 4) I smoke every day, about the same as before my pregnancy; 5) I smoke every day, and tend to smoke more than before my pregnancy.*

*W*omen who reported quitting smoking since completing the previous survey were defined as ‘ex-smokers’. Women were defined as ‘vapers’ if they reported they were currently vaping either daily, using less than daily but at least once a week, using occasionally but less than weekly, or vaping once or twice. If women reported that they smoked either daily or occasionally and did not vape (in any capacity), then they were defined as a ‘smoker’. Smokers who reported that they also vaped (in any capacity) were defined as ‘dual users’. Women who reported that they did not smoke but vaped (in any capacity) were defined as ‘exclusive vapers’.

Where follow-up surveys were missing responses to the vaping question used to define current vaping status ‘How often do you use an e-cigarette or vaping device now?’, two researchers independently reviewed the participant’s other responses to questions surrounding vaping habits (follow up survey questions; A9-A17) in order to determine vaping status.

### 2.5 Statistical analysis

To observe the pattern of vaping throughout pregnancy, we aimed to recruit at least 600 women into the cohort (Bowker et al., 2021). Analysis was conducted using Stata-SE version 15 (StataCorp LLC, College Station, TX, USA).

We described the characteristics and smoking/vaping behaviour of the women who completed a survey in early pregnancy, those who entered the cohort study and those who completed all three surveys. Using chi-squared tests for categorical variables and t-tests for continuous variables, we looked to see if there were differences between women who only completed an early pregnancy survey and women who completed a survey at each of the three time points. P values of <0.05 were deemed significant.

We then described cross sectional prevalence of vaping and smoking in early and late pregnancy and the postpartum. For women who were classified as vapers at any of the time points, we described the frequency of vaping and main reason for vaping at each time point. We presented prevalence of vaping at each time point after excluding vapers who report vaping only once or twice, to highlight the prevalence of women who regularly vape during pregnancy. We also described the frequency of vaping specifically in vapers who completed all three surveys.

We described the temporal changes in vaping status within women who completed all three surveys to explore the patterns in individuals’ smoking and vaping habits during pregnancy and postpartum. To investigate the impact of missing outcome data for smoking and vaping status in late pregnancy or the postpartum we used multiple imputation, using Stata’s mi command, based on the characteristics that were associated with non-completion of all surveys. We included the outcome variable in the model. Since some of the smoking/vaping categories had zero or very few observations, and in multiple imputation proportions could be calculated for some but not all imputed datasets due to zero observations, these rare categories were excluded from our tree diagram.

## 3 Results

### 3.1 Summary of the survey responses

**Fig. 1:**
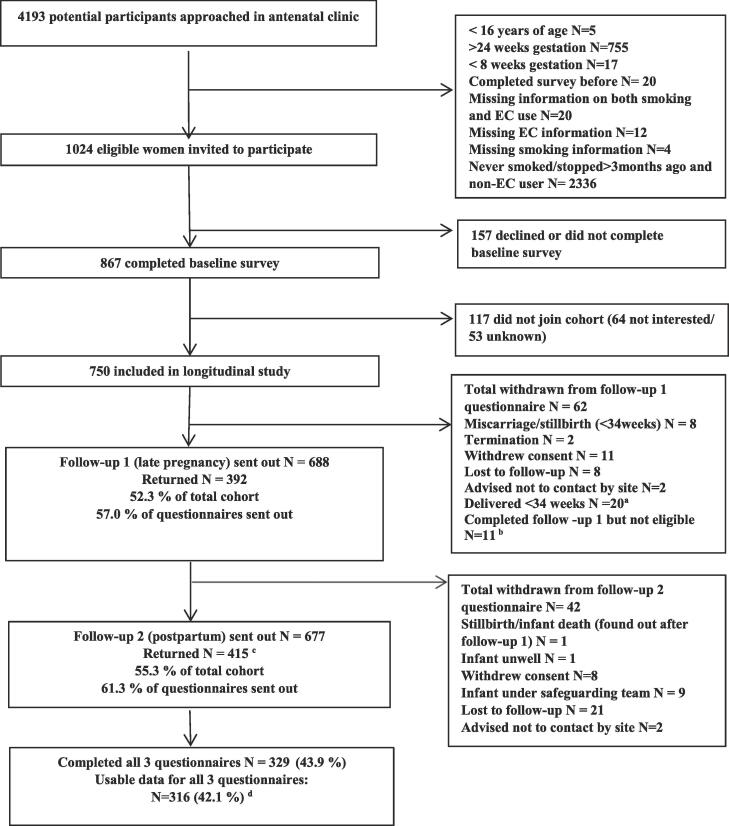
Recruitment and flow of participants through the study.

Fig. 1 summarises the survey response rates. Of 1024 eligible women, 84.6% (n = 867) completed a survey in early pregnancy (baseline) and of these 86.5% (n = 750/867) joined the cohort. Surveys were returned by 52.3% (n = 392/750) of the cohort in late pregnancy (34–38 weeks gestation) and 56.0% (n = 415/750) in postpartum (3 months after having a baby). A total of 42.1% (n = 316/750) of women completed all three surveys and had complete data on their smoking and vaping status. The characteristics of the women who completed the early pregnancy survey have been described elsewhere (Bowker et al., 2021). Supplementary Table 1 shows that compared to those who only completed the early pregnancy survey, women who completed all three surveys were significantly more likely to be ex-smokers in early pregnancy (p = 0.003), to hold higher educational qualification (p < 0.001), to have left education at a higher age (p < 0.001), to have a planned pregnancy (p < 0.001) and to report they were seriously planning on quitting smoking (p = 0.012). Women from the North and Midlands areas of England were more likely to have completed all three surveys compared with other regions (p = 0.008).

### 3.2 Cross sectional prevalence and frequency of vaping in early and late pregnancy and postpartum

Table 1 shows that in early pregnancy 15.9% (n = 119/750) of pregnant smokers or recent ex-smokers reported vaping; 12.4% (n = 93/750) were dual users and 3.5% (n = 26/750) were exclusive vapers. Reported vaping prevalence in late pregnancy was 17.8% (n = 68/383) (of which 12.5% (n = 48/383) were dual users and 5.2% (n = 20/383) exclusive vapers. In the postpartum, prevalence was 23.1% (n = 95/411) of which 14.6% (n = 60/411) were dual users and 8.5% (n = 35/411) were exclusive vapers. When vapers who reported only vaping once or twice were excluded from each time point (data not shown in table) the vaping prevalence in early pregnancy was 12.2% (n = 92/750), 13.6% (n = 52/383) in late pregnancy and 18.7% (n = 77/411) in the postpartum.

**Table 1:** Smoking and vaping status, frequency, and main reason for vaping in early and late pregnancy and the postpartum.

	Early pregnancy (n&%)	Late pregnancy (n&%)	Postpartum (n&%)
Total who completed the survey	**750**	383*	411*
Smoker	384 (51.2)	168 (43.9)	218 (53.0)
Ex-smoker	247 (32.93)	147 (38.4)	98 (23.8)
Vaper (dual and exclusive)	119 (15.9)	68 (17.8)	95 (23.1)
Frequency of vaping: Dual user			
Total	**n = 93 (12.4)**	**n = 48 (12.5)**	**n = 60 (14.6)**
*Used every day*	29 (31.2)	12 (25.0)	14 (23.3)
*Used less than daily but at least once a week*	24 (25.8)	15 (31.3)	15 (25.0)
*Used occasionally but less than weekly*	14 (15.1)	11 (22.9)	17 (28.3)
*Only used once or twice*	5 (5.4)	10 (20.8)	14 (23.3)
*Not used at all*	11 (11.8)^	0(0)	0(0)
*Missing*	10 (10.8)	0(0)	0(0)
Main reason for vaping: Dual user			
*To quit smoking*	47 (50.5)	18 (37.5)	23 (38.3)
*To cut down smoking*	28 (30.1)	15 (31.3)	17 (28.3)
*To avoid returning to smoking*	0 (0)	0(0)	5 (8.3)
*Instead of smoking around my baby*	n/a	n/a	6 (10.0)
Other **	4 (4.3)	3 (6.3)	2 (3.3)
*Missing*	12 (12.9)	12 (25.0)	7 (11.7)
Frequency of vaping: Exclusive vaper			
Total	**n = 26 (3.5)**	**n = 20 (5.2)**	**n = 35 (8.5)**
*Used every day*	17 (65.4)	15 (75.0)	27 (77.1)
*Used less than daily but at least once a week*	3 (11.5)	2 (10.0)	1 (2.9)
*Used occasionally but less than weekly*	5 (19.2)	0(0)	3 (8.6)
*Only used once or twice*	0(0)	3 (15.0)	4 (11.4)
*Not used at all*	1 (3.9)^	0(0)	0(0)
Main reason for vaping: Exclusive vaper			
*To quit smoking*	17 (65.4)	11 (55.0)	20 (57.1)
*To cut down smoking*	0(0)	0(0)	0(0)
*To avoid returning to smoking*	3 (11.5)	5 (25.0)	10 (28.6)
*Instead of smoking around my baby*	n/a	n/a	0 (0)
Other **	1 (3.9)	2 (10.0)	3 (8.7)
*Missing*	5 (19.2)	2 (10.0)	2 (5.7)

*5 women did not provide information on smoking/vaping in late pregnancy and 4 women did not provide information on smoking/vaping in the postpartum.

**‘Other’ includes: Curiosity, enjoyment, to use when I am not allowed to smoke, don’t know and other (unknown).

^ The early pregnancy survey responses contained women who stated that they vaped, but then reported having ‘not used at all’ in their response to a question about frequency of vaping.

In early pregnancy, 65.4% (n = 17/26) of exclusive vapers reported vaping daily. A total of 31.2% (n = 29/93) of dual users reported vaping daily and 25.8% (n = 24/93) vaped less than daily but at least once a week. In late pregnancy (75.0%, n = 15/20) and the postpartum (77.1%, n = 27/35) a greater proportion of exclusive vapers reported vaping daily compared with early pregnancy. Among dual users a decreased proportion reported daily vaping in late pregnancy (25.0%, n = 12/48) and postpartum (23.3%, n = 14/60) compared with early pregnancy.

When observing only women who reported vaping at all three time points, in early pregnancy most exclusive vapers reported vaping every day (66.7%, n = 4/6). By late pregnancy and the postpartum all (100%) exclusive vapers reported daily use. Dual users varied in their daily reported vaping during pregnancy, but by the postpartum only one dual user reported vaping daily (6.3%, n = 1/16).

### 3.3 Longitudinal patterns of vaping during pregnancy and the postpartum

**Fig. 2:**
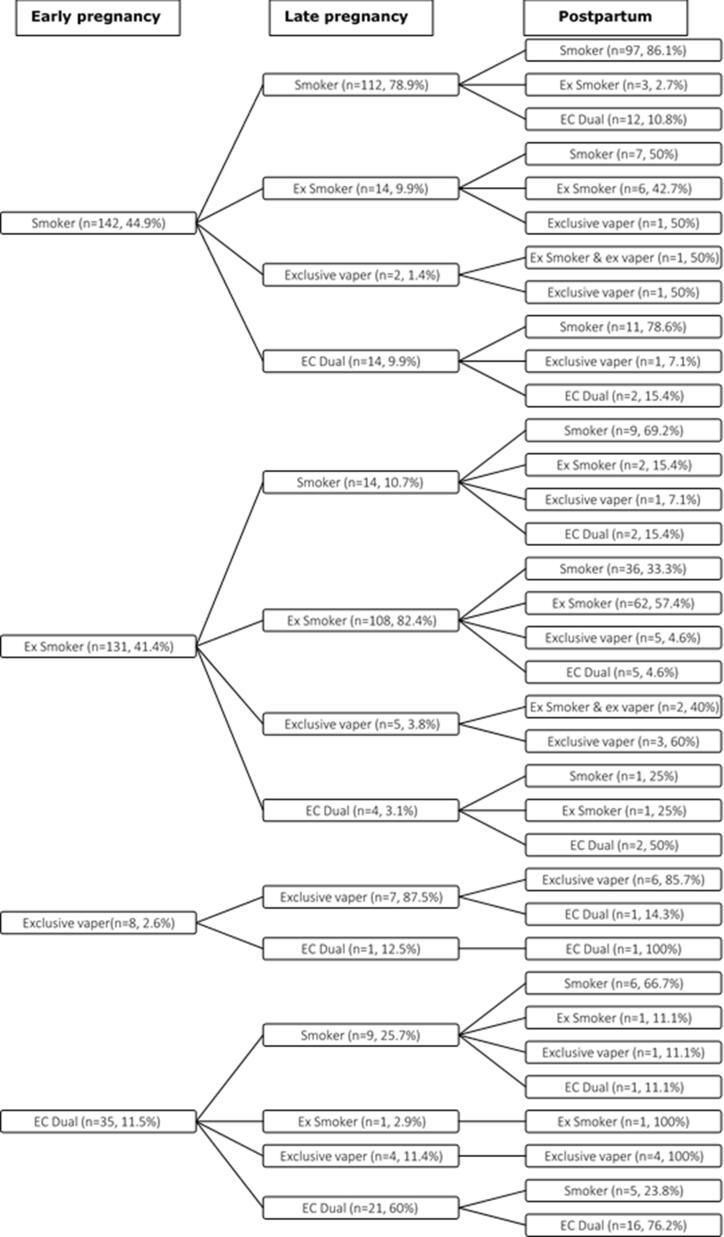
Patterns of vaping and smoking throughout pregnancy.

Fig. 2 shows the patterns of vaping and smoking behaviour within the 316 women who completed all three surveys and provided information on their smoking and vaping status. Fig. S1 shows the patterns of vaping and smoking at the three time points with missing data at follow-up imputed using multiple imputation; the patterns were similar to the non-adjusted figures.

#### 3.3.1 Patterns of women that vape in early pregnancy

In total 2.6% (n = 8/316) of women who completed all three surveys were classified as exclusive vapers in early pregnancy; most remained exclusive vapers in late pregnancy (87.5%, n = 7/8) and the postpartum (75%, n = 6/8). Exclusive vapers in early pregnancy who were no longer exclusive vapers at later time points all became dual users.

In total 11.5% (n = 35/316) of women were classified as dual users in early pregnancy; over half remained dual users (60.0%, n = 21/35) in late pregnancy, of which 76.2% (n = 16/21) were dual users in the postpartum. Some temporal changes are evident in these dual users. For example, by the postpartum around a third (31.4%, n = 11/35) of dual users in early pregnancy were exclusive smokers. Around a quarter (n = 25.7%, n = 9/35) of dual users in early pregnancy, were exclusive smokers by late pregnancy, over half of whom remained exclusive smokers in the postpartum (66.7%, n = 6/9). Nearly a quarter (23.8%, n = 5/21) of women who dual used throughout pregnancy became exclusive smokers in the postpartum. A minority of early pregnancy dual users (11.4%, n = 4/35), became exclusive vapers by late pregnancy and remained exclusive vapers in the postpartum. Only one dual user (2.9%, n = 1/35) in early pregnancy become an ex-smoker in late pregnancy and remained so in the postpartum.

#### 3.3.2 Patterns of women that do not vape in early pregnancy

There were 142 women classified as smokers in early pregnancy and 68.3% (n = 97/140), remain smokers throughout. A minority of exclusive smokers in early pregnancy were vaping in late pregnancy, either as dual users (9.9%, n = 14/142), or exclusive vapers (1.4%, n = 2/142). Those who became dual users in late pregnancy often returned to exclusive smoking in the postpartum (78.6%, n = 11/14). A minority of women who were exclusive smokers throughout pregnancy became dual users in the postpartum (10.8%, n = 12/112). Around 10% of women who were classified as ex-smokers during early and late pregnancy started vaping postpartum; 4.6% (n = 5/108) were duals users and 4.6% (n = 5/108) were exclusive vapers. A third (33.3%, n = 36/108) of ex-smokers were smoking in the postpartum.

### 3.4 Main reasons for vaping in early and late pregnancy and postpartum

The most frequently reported main reason to vape among exclusive vapers at each time point was to quit smoking: in early pregnancy 65.4% (n = 17/26), late pregnancy 55.0% (n = 11/20) and postpartum 57.1% (n = 20/35). A minority of exclusive vapers in early pregnancy reported that their main reason to vape was to avoid returning to smoking (11.5%, n = 3/26); this became a more frequent response in late pregnancy (25.0%, n = 5/20) and the postpartum (28.6%, n = 10/35). The most frequently reported main reason to vape among dual users was to quit smoking: early pregnancy 50.5% (n = 47/93), late pregnancy 37.5% (n = 18/48) and postpartum 38.3% (n = 23/60). The second most frequently reported main reason among dual users was to cut down their smoking: early pregnancy 30.1% (n = 28/93), late pregnancy 31.3% (n = 15/48) and postpartum 28.3% (n = 17/60).

## 4 Discussion

This is the first study to prospectively collect longitudinal data to describe pregnant women’s vaping throughout pregnancy and the postpartum. Our findings show that nearly 16% of pregnant smokers or ex-smokers are vaping in early pregnancy, 18% in late pregnancy and 23% in the postpartum. Most vapers during pregnancy and the postpartum report being dual users. We have also been able to report temporal changes in vaping. Vaping status among exclusive vapers in early pregnancy remained stable throughout pregnancy and the postpartum. Dual users appear less stable with around a quarter of dual users in early pregnancy becoming exclusive smokers by late pregnancy and a third exclusively smoking by the postpartum. A minority of women who were ex-smokers or smokers throughout pregnancy became vapers in the postpartum.

A limitation of this study is that we relied on self-reported data. Previous studies have shown stigma associated with both smoking and vaping during pregnancy (Katharine Bowker et al., 2018; Laura Schilling et al., 2019) and this could potentially lead to underreporting. However, there is some evidence that using self-reported smoking data during pregnancy is valid (Pickett, Rathouz, Kasza, Wakschlag, & Wright, 2005) and as there was no intervention, there was no expectation that women should stop vaping or smoking. The surveys were completed discreetly during antenatal appointments in early pregnancy (Bowker et al., 2021) and at the woman’s own discretion at follow up, enabling women to give honest responses. The participants were predominantly white British, similar to other UK cohorts of pregnant smokers (Orton et al., 2014), but we recognise that our findings may not be generalisable to other ethnicities. Our follow up rates were relatively low at 52.3% in late pregnancy and 55.3% postpartum, and only 42.1% completed all three surveys, although our multiple imputation analysis that accounted for nonresponse bias showed similar smoking and vaping patterns to the main analysis.

We have data on longitudinal patterns for a relatively small number of exclusive and dual use vapers; these low numbers are possibly a reflection of low and variable levels of vaping in pregnant populations (Whittington et al., 2018). Following a larger number of vapers over time would likely ensure a more representative understanding of vaping patterns. We defined vapers as anyone who reported vaping at any of the time points, including those who reported vaping only once or twice; we did not want to exclude infrequent vapers as we wanted to capture those experimenting with e-cigarettes. However, the prevalence of vaping after we excluded infrequent vapers showed that most vapers in our study used an e-cigarette more than once or twice. E-cigarette use may change over time and could explain the increase in proportions of those vaping in late pregnancy and the postpartum. However, when interpreting the temporal changes of vaping, consideration should be given to the highlighted differences in characteristics, such as education, between those that completed all three surveys and those that only completed the early pregnancy survey.

Exclusive vapers in early pregnancy appear less likely to return to smoking in the postpartum when compared with ex-smokers. Although we recognise the numbers of exclusive vapers were low, this pattern is similar to studies outside of pregnancy, which have shown rates of relapse to smoking in exclusive vapers is low over time (Farsalinos et al., 2014, Pasquereau et al., 2017). Exclusive vapers appear committed to vaping; the majority reported daily vaping throughout pregnancy and the postpartum. Little is known about why some pregnant women can quit smoking while vaping while others struggle; finding out more about the devices vapers use, the strengths of nicotine and adherence to e-cigarettes could aid our understanding.

Dual users commonly returned to smoking; nearly a quarter of women who reported being a dual user in early pregnancy were smoking exclusively by late pregnancy and around a third of pregnant dual users in early pregnancy were smoking exclusively in the postpartum. Dual users were less likely to report daily vaping compared to exclusive users, so it could be that their vaping habits were insufficient to assist with smoking cessation, or they were vaping as an alternative to smoking in some situations. Nevertheless, like previous studies we found dual users often reported that their primary reason for vaping was to quit smoking (Chiang et al., 2019, Fallin et al., 2016, Wagner et al., 2017). One survey, which explored vaping use before and during pregnancy, found only one pregnant woman switched from dual use before pregnancy to vaping exclusively during pregnancy (L. Schilling, Spallek, Maul, Tallarek, & Schneider, 2021). It is vital that more support is given to pregnant dual users to help them use e-cigarettes exclusively and thereby achieve their goal of smoking cessation. Although e-cigarettes are not risk free (American Lung American Lung Association, 2020, Britton et al., 2016, Froggatt et al., 2020), evidence outside of pregnancy observes health benefits among vapers who stop smoking combustible cigarettes completely (McDonnell et al., 2020, Shahab et al., 2017).

We found that nearly 11% of women who had smoked exclusively throughout pregnancy became dual users in the postpartum, and a similar proportion of women who were ex-smokers throughout pregnancy took up vaping (either exclusive or dual) in the postpartum. This could reflect women choosing to experiment with e-cigarettes as a novel product but may also be indicative of women trying to protect their new-born from second-hand smoke exposure by using e-cigarettes instead of continuing or returning to smoking in the postpartum period. Currently clinicians support pregnant smokers to stop smoking; they may also need to support dual users to stop smoking and avoid returning to smoking, and these women may have differing needs to exclusive smokers.

## 5 Conclusion

Between 16% and 23% of pregnant smokers and ex-smokers reported vaping at some point during pregnancy and the postpartum period; the majority dual use but vape with the intention to quit smoking. Temporal patterns show that the vaping habits of exclusive vapers remains stable throughout pregnancy and the postpartum. However, the vaping habits of dual users varies with a third becoming exclusive smokers by the postpartum period. Exclusive vapers appear more committed to vaping and vape daily, whereas dual users are less frequent users.

## Funding

This work was funded by Cancer Research UK (CRUK), Tobacco Advisory Group Project (Grant number C53479/A22733). CRUK had no role in the study design, collection, analysis or interpretation of the data, writing the manuscript, or the decision to submit the paper for publication.

## Author contribution

The study was conceptualized and designed by SC, SL, TC, LB, HM, MU, FN, SO, KB. KB, LP and SC were involved in planning and managing the data collection. SL, SC, KB, were involved in in the statistical analysis. KB wrote the first draft of the manuscript and all authors contributed to and have approved the final manuscript.

## Declaration of Competing Interest

Dr Hayden McRobbie has in the past 3 years received honoraria for speaking at smoking cessation meetings and attending advisory board meetings that have been organised by Pfizer. He has no relationships with the manufacturers of vaping products. All other authors declare that they have no conflicts of interest.
