# Supplementary material for: Smoking and vaping patterns during pregnancy and the postpartum: A longitudinal UK cohort survey
Source: Addict Behav. 2021 Dec;123:107050. doi: 10.1016/j.addbeh.2021.107050 (PMC8434421; doi:10.1016/j.addbeh.2021.107050)

**Supplementary Table 1: Characteristics for all women who completed the baseline survey, entered the cohort and completed all three surveys.**

|  | | **Participants who completed the baseline survey** | **Participants who entered the longitudinal cohort** | **Participants who completed all three surveys** | **Comparisons between those who completed baseline survey and all three surveys**  **P value** |
| --- | --- | --- | --- | --- | --- |
|  |  | **N=867** | **N=750** | **N=316** |  |
| **Smoking and vaping status at baseline*** | Smoker | 434 (50.1%) | 384 (51.2%) | 142 (44.9%) | 0.003 |
|  | Ex-smoker | 293 (33.8%) | 247 (32.9%) | 131 (41.5%) |  |
|  | Exclusive vaper | 33 (3.8%) | 26 (3.5%) | 8 (2.5%) |  |
|  | Dual user | 107 (12.3%) | 93 (12.4%) | 35 (11.1%) |  |
|  |  |  |  |  |  |
| **Age** | Median[1^st^ Q, 3^rd^ Q] | 26 (22-31) | 26 (22-31) | 26 (23-31) | 0.067 |
|  | Missing ± | 3 | 2 | 1 |  |
|  |  |  |  |  |  |
| **Region** | North | 290 (33.5%) | 264 (35.2%) | 115 (36.4%) | 0.008 |
|  | South | 111 (12.8%) | 87 (11.6%) | 41 (13.0%) |  |
|  | Midlands | 283 (32.6%) | 267 (35.6%) | 109 (34.5%) |  |
|  | London | 100 (11.5%) | 87 (11.6%) | 36 (11.4%) |  |
|  | Scotland | 83 (9.6%) | 45 (6.0%) | 15 (4.8%) |  |
|  |  |  |  |  |  |
| **Highest educational level** | GCSEs, similar or none | 502 (57.9%) | 439 (58.5%) | 150 (47.5%) | <0.001 |
|  | A levels/ degree, similar or above | 348 (40.1) | 304 (40.5%) | 163 (50.9%) |  |
|  | Missing ± | 17 (2.0%) | 7 (0.9%) | 3 (1.0%) |  |
|  |  |  |  |  |  |
|  | 16 and under | 415 (47.9%) | 368 (49.1%) | 131 (41.5%) | <0.001 |
| **Age left education** | 17 and above | 408 (47.1%) | 352 (46.9%) | 175 (55.4%) |  |
|  | Still in education | 25 (2.9%) | 21 (2.8%) | 9 (2.9%) |  |
|  | Missing ± | 19 (2.2%) | 9 (1.2%) | 1 (0.3%) |  |
|  |  |  |  |  |  |
| **Ethnicity** | White British | 759 (87.5%) | 662 (88.3%) | 274 (86.7%) | 0.286 |
|  | Other | 63 (11.1%) | 84 (11.2%) | 40 (12.7%) |  |
|  | Missing ± | 12 (1.4%) | 4 (0.5%) | 2 (0.6%) |  |
|  |  |  |  |  |  |
| **Gestation at recruitment (weeks)** | First trimester | 399 (46.0%) | 337 (44.9) | 139 (44.0) | 0.270 |
|  | Second trimester | 460 (53.1) | 413 (55.1) | 177 (56.0) |  |
|  | Don’t know/missing | 8 (0.9) | 0 | 0 |  |
|  |  |  |  |  |  |
| **Previous pregnancy** | Not been pregnant before (no) | 288 (33.2%) | 248 (33.1%) | 107 (33.9%) | 0.861 |
|  | Been pregnant before (yes) | 561 (64.7%) | 490 (65.3%) | 205 (64.9%) |  |
|  | Missing ± | 18 (2.1%) | 12 (1.6%) | 4 (1.3%) |  |
|  |  |  |  |  |  |
| **If yes – smoked in previous pregnancy** |  | N=561 | N=490 | N=205 |  |
|  | Yes | 345 (61.5%) | 307 (62.7%) | 122 (60.0%) | 0.435 |
|  | No | 197 (35.1%) | 165 (33.7%) | 75 (36.6%) |  |
|  | Don’t remember | 12 (2.1%) | 12 (2.5%) | 6 (2.9%) |  |
|  | Missing ± | 7 (1.3%) | 6 (0%) | 2 (1.0%) |  |
|  |  |  |  |  |  |
| **Planned pregnancy** | Yes | 292 (33.7%) | 252 (33.6%) | 130 (41.1%) | <0.001 |
|  | No | 548 (63.2%) | 480 (64.0%) | 177 (56.0%) |  |
|  | Missing ± | 27 (3.1%) | 18 (2.4%) | 9 (2.9%) |  |
|  |  |  |  |  |  |
| **Tried to stop smoking since becoming pregnant** | Yes | 639 (73.7%) | 554 (73.9%) | 228 (72.2%) | 0.179 |
|  | No | 142 (16.4%) | 127 (16.9%) | 53 (16.8%) |  |
|  | Stop smoking before pregnancy | 61 (7.0%) | 53 (7.1%) | 27 (8.5%) |  |
|  | Missing ± | 25 (2.7%) | 16 (2.1%) | 8 (2.5%) |  |
|  | |  |  |  |  |
| **Current smokers only** | | **541** | **477** | **177** |  |
| **Last smoked** | In last 24 hours | 470 (86.9%) | 419 (87.8%) | 154 (87.0%) | 0.487 |
|  | Over 24 hours | 59 (10.9%) | 50 (10.5%) | 22 (12.4%) |  |
|  | Missing ± | 12 (2.2%) | 8 (1.7%) | 1 (0.6%) |  |
|  |  |  |  |  |  |
| **Heaviness of Smoking Index** | Low dependence (0-2) | 345 (63.8%) | 306 (64.2%) | 113 (63.8%) | 0.465 |
|  | Moderate/high dependence (3-6) | 158 (29.2%) | 143 (30.0%) | 57 (32.2%) |  |
|  | Missing ± | 38 (7.0%) | 28 (5.9%) | 7 (4.0%) |  |
|  |  |  |  |  |  |
| **Cigarettes smoked per day** | 0-10 | 387 (71.5%) | 341 (71.5%) | 123 (69.5%) | 0.476 |
|  | ≥ 11 | 128 23.7%) | 116 (24.3%) | 48 (27.1%) |  |
|  | Missing ± | 26 (4.8%) | 20 (4.2%) | 6 (3.4%) |  |
|  |  |  |  |  |  |
| **Seriously planning to quit smoking** | Yes | 362 (66.9%) | 326 (68.3%) | 131 (74.0%) | 0.012 |
|  | No | 128 (23.7%) | 112 (23.5%) | 36 (20.3%) |  |
|  | Missing ± | 51 (9.4%) | 39 (8.2%) | 10 (5.7%) |  |
| **± missing excluded from chi squared tests** | | | | |  |

**Supplementary Figure 1: smoking and vaping patterns using multiple imputation.**


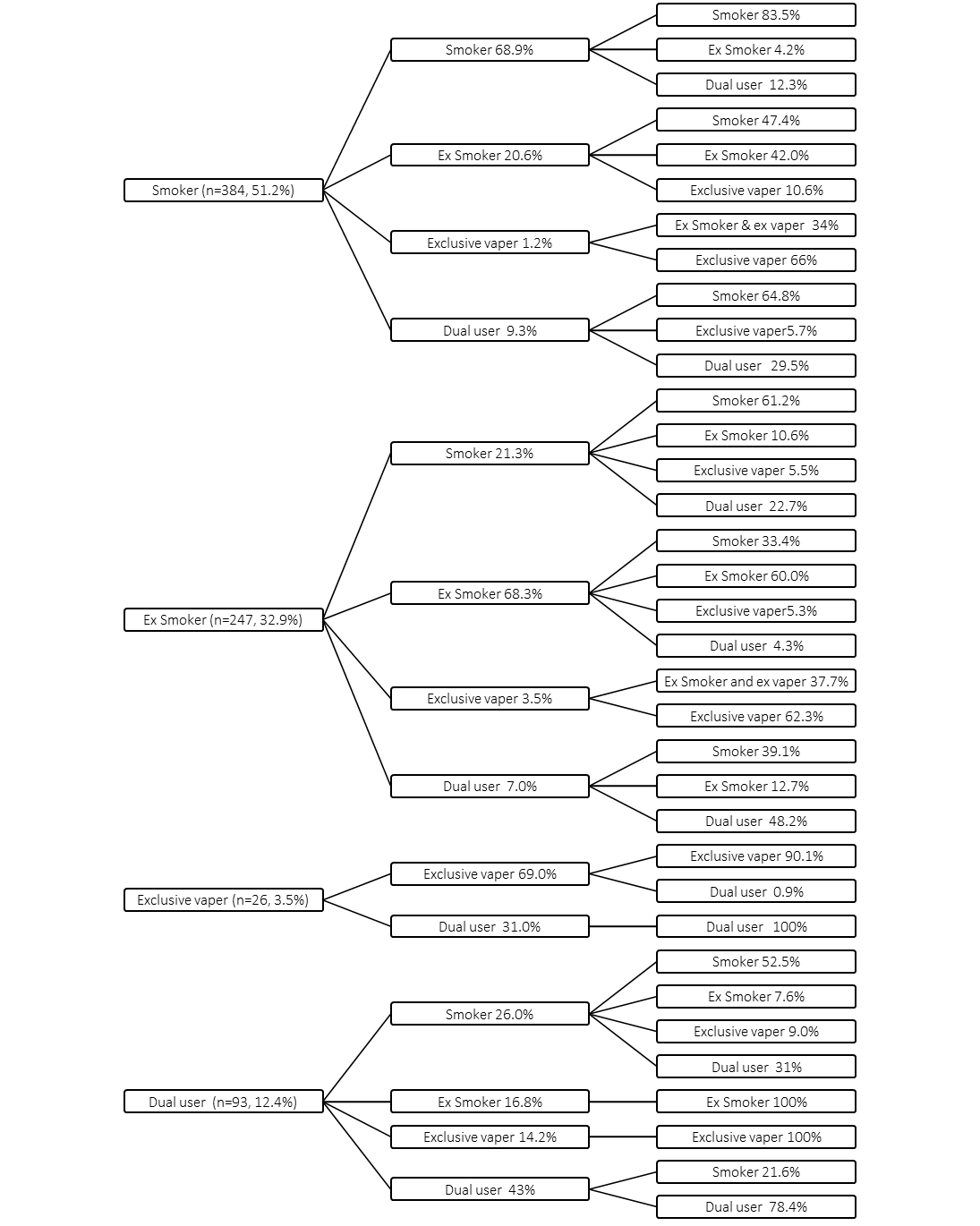

Supplement: Supplementary data 1 [file mmc1.docx]
