# Supplementary material for: Smoking and vaping patterns during pregnancy and the postpartum: A longitudinal UK cohort survey
Source: Addict Behav. 2021 Dec;123:107050. doi: 10.1016/j.addbeh.2021.107050 (PMC8434421; doi:10.1016/j.addbeh.2021.107050)
Supplement: Supplementary data 2 [file mmc2.pdf]

The University of Nottingham

UNITED KINGDOM · CHINA · MALAYSIA

Trust LOGOs to be added

Researchers' use only

Participant ID number			–				–			
	Site number			Participant number				Initials		
Date returned			/			/				
	D	D		M	M		Y	Y	Y	Y
Researcher's initials										

Pregnancy lifestyle survey 2017

Pregnant women's attitudes towards smoking and electronic cigarettes

BASELINE QUESTIONNAIRE
Final Version: 3.1
Version date: 27th June 2017

Dear Madam,

We are looking at ways to improve the health and lifestyle of pregnant women and their babies. We are therefore inviting ALL women who come to the antenatal clinic to answer a few questions. These include questions about smoking and electronic cigarette use, but we would like as many women as possible to answer the first few questions, even if you have never smoked or used e-cigarettes, so that we can see how common these are in *all* pregnant women.

Your answers to these questions will be used in our research, but will be anonymous and your name will not appear anywhere in our reports.

The questionnaire is optional and not related to your clinical care. Whether or not you decide to complete it, this will not affect the care you receive.

Thank you for reading this and for your help.

Best wishes

Dr Sue Cooper
Division of Primary Care, University of Nottingham
Nottingham NG7 2RD

Participant ID number

		–				–		

SCREENING QUESTIONS

Your answers to the questions on this page and the next will determine whether you should complete the rest of the questionnaire. Please read the instructions carefully. Thank you.

S1 Have you completed this questionnaire before? (e.g. on a previous visit to the antenatal clinic or for a scan)

❑ Yes ❑ No ❑ Don't know

If you ticked **Yes**, please **hand back the questionnaire** - you are finished – thank you. Otherwise please continue

S2 Approximately, how many weeks pregnant are you?

☐☐ weeks ❑ Don't know

S3 How old are you?

☐☐ Years of age

If you are:

a) less than 8 weeks or more than 24 weeks pregnant, **or**
b) you are under 16 years old

Then please **hand back the questionnaire** - you are finished – thank you.
Otherwise, please answer the next 2 questions.

Participant ID number | | | – | | | – | | |

The next question is about **electronic cigarettes**, often called **e-cigarettes**.

They are also sometimes called e-pen, e-shisha, vape pens, vape mods, box mods or ENDs. E-cigarettes produce a vapour that looks like smoke but, unlike normal cigarettes, they are not lit with a flame and do not heat or burn tobacco. Here are some examples of what they look like:

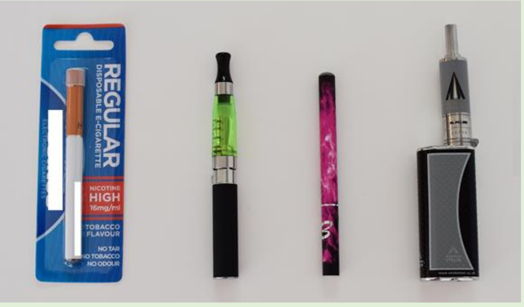

S4 Please tick the box below next to the statement that best describes your use of **e-cigarettes** <u>right now</u>

1 ❑ I have never heard of e-cigarettes and have never tried them

2 ❑ I have heard of e-cigarettes, but have never tried them

3 ❑ I have tried e-cigarettes, but do not use them now

4 ❑ I have tried e-cigarettes and still use them, but not every day

5 ❑ I have tried e-cigarettes and still use them every day

S5 We would also like to know about smoking. Please tick the box below next to the statement that best describes your **smoking** <u>right now</u>

1 ❑ I have never smoked

2 ❑ I completely stopped smoking **more than** 3 months before finding out I was pregnant

3 ❑ I completely stopped smoking at some time **in** the 3 months before finding out I was pregnant

4 ❑ I completely stopped smoking after I found out I was pregnant

5 ❑ I smoke occasionally, but not every day now I am pregnant

6 ❑ I smoke every day, but have cut down during my pregnancy

7 ❑ I smoke every day, about the same as before my pregnancy

8 ❑ I smoke every day, and tend to smoke more than before my pregnancy

Participant ID number | | | – | | | | – | | |

Have you ticked one or more of the coloured boxes in either question S4 and/or S5 above? If **YES**, please **continue to read below**.

If **NO** (you **did not** tick **any** coloured boxes in S4 or S5), please **hand back the questionnaire** - you are finished – thank you.

(If you are not sure whether you should be filling in the rest of the questionnaire please ask [the research midwife])

From your answers, so far, we are interested in finding out more about your views on e-cigarettes, smoking and pregnancy. We would be very grateful if you would read the following information sheet that gives more details about the study we are carrying out.

If you want to ask any questions either before or after you read this then please ask to speak to [the research midwife] who is [in the clinic waiting area].

After you have read the information sheet, if you are happy to continue, you can complete the questionnaire. Once you have finished this, we will give you a **£10 gift voucher** to thank you for your help.

PATIENT INFORMATION SHEET INSERTED HERE

Participant ID number | | | – | | | | – | | |

YOUR VIEWS AND EXPERIENCE OF E-CIGARETTES

In this section, **we would like you to answer some questions about your experience of and views on e-cigarettes – even if you haven't used them.**

Some questions are for everyone and others are for particular individuals (e.g., those currently using e-cigarettes).

Everyone should answer A1 to A9 below. After that, please follow the instructions about which questions to answer. Thank you.

A1 How often did you use an e-cigarette or vaping device during each of the following times? (Please tick one box for each time period)

	Not used at all	Only used once or twice	Used occasionally, but less than weekly	Used less than daily, but at least once a week	Used every day
In the 3 months before you became pregnant	❑	❑	❑	❑	❑
In the first 2 months of your pregnancy	❑	❑	❑	❑	❑
Now	❑	❑	❑	❑	❑

A2 Does anyone you know ***use*** an e-cigarette? (tick as many as apply)

❑ Yes, my partner ❑ Yes, a family member or friend ❑Yes, someone else I know ❑ No

A3 Do you know anyone who has used an e-cigarette ***in pregnancy***? (tick as many as apply)

❑ Yes, a family member or friend ❑ Yes, someone else I know ❑No

Participant ID number | | | – | | | – | | |

A4 Does anyone you know think ***you*** should use an e-cigarette in pregnancy instead of smoking cigarettes? (tick as many as apply)

❑Yes, my partner
❑ Yes, another family member or friend
❑ Yes, a health professional (e.g., midwife, stop smoking advisor, GP)
❑Yes, someone else I know
❑ No
❑ I have not discussed this with anyone

A5 We would like to know your views on using e-cigarettes in pregnancy.

Please indicate how much you agree or disagree with each statement below.

(Select one answer per row)

	**Strongly disagree**	**Moderately disagree**	**Slightly disagree**	**Neither agree nor disagree**	**Slightly agree**	**Moderately agree**	**Strongly agree**
E-cigarettes should be promoted to help pregnant women stop smoking	❑	❑	❑	❑	❑	❑	❑
I'd be more likely to use an e-cigarette in pregnancy if a health professional recommended it	❑	❑	❑	❑	❑	❑	❑
I would feel comfortable using an e-cigarette in public while I am pregnant	❑	❑	❑	❑	❑	❑	❑
I would feel comfortable using an e-cigarette in public *after* having my baby	❑	❑	❑	❑	❑	❑	❑
E-cigarettes should only be used in the home or private places	❑	❑	❑	❑	❑	❑	❑
I would take part in a research study that involved using an e-cigarette whilst I am pregnant	❑	❑	❑	❑	❑	❑	❑

Participant ID number | | | – | | | | – | | |

A6 We would like to know your views on the safety of e-cigarettes in pregnancy.

Please indicate how much you agree or disagree with each statement below.

Select one answer per row

	**Strongly disagree**	**Moderately disagree**	**Slightly disagree**	**Neither agree nor disagree**	**Slightly agree**	**Moderately agree**	**Strongly agree**
Using e-cigarettes in pregnancy harms my baby	❑	❑	❑	❑	❑	❑	❑
Using e-cigarettes in pregnancy is as safe as using nicotine patches	❑	❑	❑	❑	❑	❑	❑
Using e-cigarettes in pregnancy is much less harmful than smoking tobacco cigarettes	❑	❑	❑	❑	❑	❑	❑
Nicotine is harmful to my unborn baby	❑	❑	❑	❑	❑	❑	❑
E-cigarettes should only be available on prescription	❑	❑	❑	❑	❑	❑	❑

Participant ID number | | | – | | | | – | | | |

A7 Below are some reasons people may give for using e-cigarettes ***in pregnancy***.

In the first column, please tick the reason that ***you*** think is the ***most important***. In the second column, tick any other reasons that you think are important for using e-cigarettes in pregnancy.

	Most important reason for using e-cigarettes **(tick one only)**	Other reasons for using e-cigarettes **(tick any you think are important)**
To help me quit smoking	❑	❑
To help me cut down the number of cigarettes	❑	❑
They are healthier than tobacco cigarettes	❑	❑
They are cheaper than tobacco cigarettes	❑	❑
They are less harmful to others around me than tobacco cigarettes	❑	❑
They feel similar to smoking	❑	❑
They look fashionable / stylish	❑	❑
They taste and smell nicer than tobacco cigarettes	❑	❑
I can use them in places where smoking is banned	❑	❑
It is more acceptable to be seen using e-cigarettes than smoking tobacco	❑	❑
Other reason (please state) ………………………………………………………………………………………	❑	❑

A8 Below are some reasons people may give for **NOT** using e-cigarettes ***in pregnancy***.

In the first column, please tick the reason that ***you*** think is the ***most important***. In the second column, tick any other reasons that you think are important for not using e-cigarettes in pregnancy.

	Most important reason for NOT using e-cigarettes **(tick one only)**	Other important reasons for NOT using e-cigarettes **(tick any you think are important)**
Bad stories in the press or on social media put me off them	❑	❑
Family or friends tell me not to use them	❑	❑
They don't get rid of the 'smoking habit'	❑	❑
They don't get rid of nicotine addiction	❑	❑
They don't satisfy my cravings for cigarettes	❑	❑
There isn't enough research on them	❑	❑
I'd be too embarrassed to use them	❑	❑
I'd rather try other ways to quit smoking, such as nicotine patches	❑	❑
I'm worried about side effects	❑	❑
I get different messages from different people so I'm confused	❑	❑
Other reason (please state) …………………………………………………………………………………	❑	❑

A9 How likely are you to try or continue to use e-cigarettes in the future?

i. While you are still pregnant:

- ❑ Very likely
- ❑ Somewhat likely
- ❑ Neither likely nor unlikely (neutral)
- ❑ Somewhat unlikely
- ❑ Very unlikely
- ❑ I don't know

ii. Once your baby is born:

- ❑ Very likely
- ❑ Somewhat likely
- ❑ Neither likely nor unlikely (neutral)
- ❑ Somewhat unlikely
- ❑ Very unlikely
- ❑ I don't know

If you **currently use e-cigarettes, even if this is not every day**, please answer **Questions A10 to A17**

If you **do not currently use e-cigarettes**, please **go to Question B1 on page 14**

A10 How soon after you wake up do you first use your e-cigarette?

- ❑ Within 5 minutes
- ❑ 31-60 minutes
- ❑ 6-30 minutes
- ❑ After 60 minutes

A11 What type of e-cigarette or vaping device do you use the most?
SELECT ONE OPTION

❑ A disposable e-cigarette or vaping device (non–rechargeable)

❑ A rechargeable e-cigarette or vaping device that uses replaceable pre-filled cartridges

❑ A rechargeable e-cigarette or vaping device with a tank that you can refill with fluids

❑ A rechargeable modular system that you refill with liquids (you use your own combination of separate parts: battery, atomiser, fluid etc.)

❑ Don't know

A12 What strength of e-liquid / fluid / juice / cartridge do you usually use?
SELECT ONE OPTION

❑ Zero (contains no nicotine)

❑ Low (up to 6mg (0.6%) nicotine)

❑ Medium (between 7 and 12mg (0.7-1.2%) of nicotine)

❑ High (between 13 and 20mg (1.3-2.0%) of nicotine)

❑ Very high (more than 20mg (2.0%) of nicotine)

❑ Don't know

A13 Please try to estimate how much on average you use your e-cigarette per day

Number of mls of fluid / juice	
Number of cartridges	
Don't know	❑

A14 Which is your preferred e-cigarette flavour category?
SELECT ONE OPTION

❑ Tobacco

❑ Tobacco menthol, menthol or mint

❑ Some other flavour like fruit, candy, alcohol, coffee, vanilla etc.

❑ No flavour

❑ Don't know

Participant ID number

		–				–			

A15 What is your main reason for using e-cigarettes? (**SELECT ONE OPTION**)

- ❑ To quit smoking
- ❑ To cut down smoking
- ❑ To use when I cannot or am not allowed to smoke
- ❑ To avoid returning to smoking
- ❑ Because I enjoy it
- ❑ Curiosity / just wanted to try them
- ❑ Some other reason. Please state:
- ❑ Don't know

A16 When do you tend to use an e-cigarette? (**Please tick all that apply**)

- ❑ When I get a craving to smoke or vape
- ❑ When I experience withdrawal symptoms like feeling restless, or irritable
- ❑ When I am feeling low
- ❑ When socialising
- ❑ When I see someone smoking
- ❑ First thing in the morning
- ❑ After a meal
- ❑ When I'm angry or stressed
- ❑ When I'm bored
- ❑ When I'm somewhere I can't smoke cigarettes
- ❑ When I don't want to or can't go outside to smoke (e.g. bad weather)
- ❑ Other. Please state:
- ❑ None of the above

Participant ID number | | | – | | | – | | |

A17 Please indicate how much you agree or disagree with each statement below.

(Select one answer per row)

	**Strongly disagree**	**Moderately disagree**	**Slightly disagree**	**Neither agree nor disagree**	**Slightly agree**	**Moderately agree**	**Strongly agree**
Using an e-cigarette is as satisfying as smoking	❑	❑	❑	❑	❑	❑	❑
Using an e-cigarette reduces / reduced my urge to smoke	❑	❑	❑	❑	❑	❑	❑
Using an e-cigarette has helped me to quit smoking	❑	❑	❑	❑	❑	❑	❑
Using an e-cigarette has helped me to reduce the number of cigarettes I smoke	❑	❑	❑	❑	❑	❑	❑

YOUR SMOKING BEHAVIOUR AND BELIEFS

Everyone should answer this section (**Questions B1 to B5**)

B1 When did you last smoke any cigarettes or tobacco (even a puff)?

❑ In the last 24 hours
❑ 1-6 days ago
❑ 7-30 days ago
❑ 1-2 months ago
❑ 2-3 months ago
❑ More than 3 months ago
❑ I have never smoked cigarettes or tobacco

B2 Since finding out you were pregnant, have you tried to stop smoking?

❑ Yes
❑ No
❑ I stopped smoking before I became pregnant
❑ I have never smoked

B3 Have you been pregnant before?

❑Yes ❑ No

If Yes, did you smoke at all during your last pregnancy?

❑Yes ❑No ❑ I don't remember

B4 This time, were you planning to get pregnant or was it a surprise?

❑ Planning ❑Was a surprise

Participant ID number | | | – | | | | – | | |

B5 If you currently smoke, or have stopped smoking, please answer each of the following questions by circling the appropriate number. **Please circle one number per question.**

	**Not at all**	**A little**	**Moderately**	**Very much**	**Extremely**
How **determined** are you to stop smoking until your baby is born?	1	2	3	4	5
How **confident** are you that you can stop smoking until your baby is born?	1	2	3	4	5
How **determined** are you to stop smoking for good?	1	2	3	4	5
How **confident** are you that you can stop smoking for good?	1	2	3	4	5

If you **SMOKE EVERY NOW & AGAIN or MORE OFTEN THAN THIS** please answer **questions B6 to B8**

If you **DO NOT SMOKE AT THE MOMENT** go to question **C1** on **page 17**

B6 Approximately how many cigarettes do you smoke each day?

- ❑ 0-5
- ❑ 6-10
- ❑ 11-15
- ❑ 16-20
- ❑ 21-30
- ❑ 31 or more

B7 How soon after you wake up do you smoke your first cigarette of the day?

- ❑ Within 5 minutes
- ❑ 6-30 minutes
- ❑ 31-60 minutes
- ❑ After 60 minutes

B8 Are you seriously planning to quit smoking?

- ❑ Yes, within the next 2 weeks
- ❑ Yes, within the next 30 days
- ❑ Yes, within the next 3 months
- ❑ No, I am not seriously planning to quit

Participant ID number | | | – | | | | – | | | |

ABOUT YOU

Everyone should answer this section (**Questions C1 to C3**)

C1 At what age did you leave/finish full time education?

[ ][ ] Years of age

❑ I am still in education

C2 Which of the following qualifications do you have?

❑ None

❑ GCSEs or similar (e.g. level 1 diploma or NVQ level 1/2)

❑ A-levels/AS-levels or similar (e.g. level 2 diploma or NVQ level 3)

❑ Degree or similar (e.g. diploma in higher education or NVQ level 4)

❑ Other (please specify) [ ]

C3 How would you describe your ethnic group?

❑ White British

❑ White Irish

❑ Any other White background

❑ Mixed - White and Black Caribbean

❑ Mixed - White and Asian

❑ Any other mixed background

❑ Asian or Asian British - Indian

❑ Asian or Asian British - Pakistani

❑ Asian or Asian British - Bangladeshi

❑ Any other Asian background

❑ Black or Black British Caribbean

❑ Black or Black British African

❑ Any other Black background

❑ Chinese

❑ Arab

❑ Other ethnic group. Please specify:

[ ]

Participant ID number

		–				–			

Thank you for completing the questionnaire.

Please hand this to [the research midwife] in clinic who will give you a £10 shopping voucher and may talk with you about the rest of the study.

If you can't find [the research midwife], then please speak to one of the reception staff in clinic who will, if necessary, take your contact details so that [the research midwife] can get in touch with you later.

If you haven't had time to complete the entire questionnaire today, or if you want some more time to think about it, then please ask [the research midwife] or reception staff for a return envelope. Once you have decided, you can return the questionnaire along with your completed details on the attached sheet, and [a researcher from the University of Nottingham] will contact you.

Thank you for your time today. We are very grateful for your help.

Participant ID number

		–				–			

CONTACT DETAILS

If you would like to continue to take part in the study, or you would like to discuss this further, then please complete your personal details below.

[A researcher from the University of Nottingham] will contact you as soon as possible

Name	
Address	
Telephone	Day: Evening:
	Mobile:
Best time to contact	
Email address	
