# Supplementary material for: Smoking and vaping patterns during pregnancy and the postpartum: A longitudinal UK cohort survey
Source: Addict Behav. 2021 Dec;123:107050. doi: 10.1016/j.addbeh.2021.107050 (PMC8434421; doi:10.1016/j.addbeh.2021.107050)
Supplement: Supplementary data 3 [file mmc3.pdf]

University of Nottingham
UK | CHINA | MALAYSIA

Researchers' use only

Participant ID number			–				–			
	Site number			*Participant number*					*Initials*	
Date returned			/			/				
	D	D		M	M		Y	Y	Y	Y
Researcher's initials										

Pregnancy lifestyle survey 2017

Pregnant women's attitudes towards smoking and electronic cigarettes

FOLLOW UP 1 QUESTIONNAIRE
Final Version: 1.0
Version date: 20th July 2017

Please complete this questionnaire **within the next 2 weeks** and then return it in the envelope provided (no stamp required). Once you have completed and returned the questionnaire, we will send you a £10 shopping voucher to thank you for your time and support.

The information you give us will be anonymous and only used by the Pregnancy Lifestyle Survey researchers.

If you have any questions or concerns about this questionnaire, please call the Smoking and Pregnancy Research Office on 0115 XXX XXXX

Thank you for your help

Today's date (please enter the date you completed this):

………… /…………… /…………

dd / mm / yyyy

Participant ID number | | | – | | | | – | | |

YOUR EXPERIENCE OF E-CIGARETTES

In this section, **we would like you to answer some questions about your experiences of and views on e-cigarettes – even if you haven't used them.**

Some questions are for everyone and others are for particular individuals (e.g., those currently using e-cigarettes).

Everyone should answer A1 to A8 below. After that, please follow the instructions about which questions to answer. Thank you.

A1 How often did you use an e-cigarette or vaping device during each of the following times? (Please tick one box for each time period)

	Not used at all	Only used once or twice	Used occasionally, but less than weekly	Used less than daily, but at least once a week	Used every day
Since you completed the first questionnaire when you joined this study	❑	❑	❑	❑	❑
Now	❑	❑	❑	❑	❑

A2 Do you know anyone who has used an e-cigarette ***in pregnancy***? (tick as many as apply)

❑ Yes, a family member or friend ❑ Yes, someone else I know ❑No

A3 Does anyone you know think ***you*** should use an e-cigarette in pregnancy instead of smoking cigarettes? (tick as many as apply)

❑Yes, my partner ❑ Yes, another family member or friend ❑ Yes, a health professional (e.g., midwife, stop smoking advisor, GP)

❑Yes, someone else I know ❑ No ❑ I have not discussed this with anyone

Participant ID number | | | – | | | | – | | |

A4 We would like to know your views on using e-cigarettes in pregnancy.

Please indicate how much you agree or disagree with each statement below.

(Select one answer per row)

	**Strongly disagree**	**Moderately disagree**	**Slightly disagree**	**Neither agree nor disagree**	**Slightly agree**	**Moderately agree**	**Strongly agree**
E-cigarettes should be promoted to help pregnant women stop smoking	❑	❑	❑	❑	❑	❑	❑
I'd be more likely to use an e-cigarette in pregnancy if a health professional recommended it	❑	❑	❑	❑	❑	❑	❑
I would feel comfortable using an e-cigarette in public while I am pregnant	❑	❑	❑	❑	❑	❑	❑
I would feel comfortable using an e-cigarette in public *after* having my baby	❑	❑	❑	❑	❑	❑	❑
E-cigarettes should only be used in the home or private places	❑	❑	❑	❑	❑	❑	❑
I would take part in a research study that involved using an e-cigarette whilst I am pregnant	❑	❑	❑	❑	❑	❑	❑

Participant ID number | | | – | | | | – | | | |

A5 We would like to know your views on the safety of e-cigarettes in pregnancy.

Please indicate how much you agree or disagree with each statement below.

(Select one answer per row)

	**Strongly disagree**	**Moderately disagree**	**Slightly disagree**	**Neither agree nor disagree**	**Slightly agree**	**Moderately agree**	**Strongly agree**
Using e-cigarettes in pregnancy harms my unborn baby	❑	❑	❑	❑	❑	❑	❑
Using e-cigarettes in pregnancy is as safe as using nicotine patches	❑	❑	❑	❑	❑	❑	❑
Using e-cigarettes in pregnancy is much less harmful than smoking tobacco cigarettes	❑	❑	❑	❑	❑	❑	❑
Nicotine is harmful to my unborn baby	❑	❑	❑	❑	❑	❑	❑
E-cigarettes should only be available on prescription	❑	❑	❑	❑	❑	❑	❑

Participant ID number

		–				–			

A6 Below are some reasons people may give for using e-cigarettes ***in pregnancy***.

In the first column, please tick the reason that you think is the ***most important***. In the second column, tick any other reasons that you think are important for using e-cigarettes in pregnancy.

	Most important reason for using e-cigarettes **(tick one only)**	Other reasons for using e-cigarettes **(tick any you think are important)**
To help me quit smoking	❑	❑
To help me cut down the number of tobacco cigarettes	❑	❑
They are healthier than tobacco cigarettes	❑	❑
They are cheaper than tobacco cigarettes	❑	❑
They are less harmful to others around me than tobacco cigarettes	❑	❑
They feel similar to smoking	❑	❑
They look fashionable / stylish	❑	❑
They taste and smell nicer than tobacco cigarettes	❑	❑
I can use them in places where smoking is banned	❑	❑
It is more acceptable to be seen using e-cigarettes than smoking tobacco cigarettes	❑	❑
Other reason (please state) …………………………………………………………………………………………	❑	❑

Participant ID number | | | – | | | – | | |

A7 Below are some reasons people may give for **NOT** using e-cigarettes ***in pregnancy***.

In the first column, please tick the reason that you think is the ***most important***. In the second column, tick any other reasons that you think are important for not using e-cigarettes in pregnancy.

	Most important reason for NOT using e-cigarettes **(tick one only)**	Other important reasons for NOT using e-cigarettes **(tick any you think are important)**
Bad stories in the press or on social media put me off them	❑	❑
Family or friends tell me not to use them	❑	❑
They don't get rid of the 'smoking habit'	❑	❑
They don't get rid of nicotine addiction	❑	❑
They don't satisfy my cravings for tobacco cigarettes	❑	❑
There isn't enough research on them	❑	❑
I'd be too embarrassed to use them	❑	❑
I'd rather try other ways to quit smoking, such as nicotine patches	❑	❑
I'm worried about side effects	❑	❑
I get different messages from different people so I'm confused	❑	❑
Other reason (please state) ………………………………………………………………………………………	❑	❑

Participant ID number

		–				–			

A8 How likely are you to try or use e-cigarettes in the future?

i. *While you are still pregnant:*

- ❑ Very likely
- ❑ Somewhat likely
- ❑ Neither likely nor unlikely (neutral)
- ❑ Somewhat unlikely
- ❑ Very unlikely
- ❑ I don't know

ii. *Once your baby is born:*

- ❑ Very likely
- ❑ Somewhat likely
- ❑ Neither likely nor unlikely (neutral)
- ❑ Somewhat unlikely
- ❑ Very unlikely
- ❑ I don't know

If you **currently use e-cigarettes, even if this is not every day**, please answer **Questions A9 to A17**

If you **do not currently use e-cigarettes**, please **go to Question B1 on page 11**

A9 How soon after you wake up do you first use your e-cigarette?

- ❑ Within 5 minutes
- ❑ 31-60 minutes
- ❑ 6-30 minutes
- ❑ After 60 minutes

Participant ID number

		–				–			

A10 What type of electronic cigarette or vaping device do you use the most?
SELECT ONE OPTION

❑ A disposable e-cigarette or vaping device (non–rechargeable)

❑ A rechargeable e-cigarette or vaping device that uses replaceable pre-filled cartridges

❑ A rechargeable e-cigarette or vaping device with a tank that you can refill with fluids

❑ A rechargeable modular system that you refill with liquids (you use your own combination of separate parts: battery, atomiser, fluid etc.)

❑ Don't know

A11 What strength of e-liquid / fluid / juice / cartridge do you usually use?
SELECT ONE OPTION

❑ Zero (contains no nicotine)

❑ Low (up to 6mg (0.6%) nicotine)

❑ Medium (between 7 and 12mg (0.7-1.2%) of nicotine)

❑ High (between 13 and 20mg (1.3-2.0%) of nicotine)

❑ Very high (more than 20mg (2.0%) of nicotine)

❑ It contains nicotine, but I don't know what strength

❑ I don't know if it contains nicotine

A12 Please try to estimate how much on average you use your e-cigarette per day

Number of mls of fluid / juice	
Number of cartridges	
Don't know	❑

A13 Have you changed how much you use e-cigarettes **since you completed the first questionnaire when you joined the study**?
SELECT ONE OPTION

❑ Yes, I tend to use them **less** now

❑ Yes, I tend to use them **more** now

❑No, I tend to use them about the **same** now

❑ Not applicable. I wasn't using an e-cigarette when I joined the study

❑ Don't know / can't remember

		–				–			

A14 Which is your preferred e-cigarette flavour category?
SELECT ONE OPTION

❑ Tobacco

❑ Tobacco menthol, menthol or mint

❑ Some other flavour like fruit, candy, alcohol, coffee, vanilla etc.

❑ No flavour

❑ Don't know

A15 What is your main reason for using an e-cigarette? (**SELECT ONE OPTION**)

❑ To quit smoking

❑ To cut down smoking

❑ To use when I cannot or am not allowed to smoke

❑ To avoid returning to smoking

❑ Because I enjoy it

❑ Curiosity / just wanted to try them

❑ Some other reason (please state) ____________________

❑ Don't know

Participant ID number | | | – | | | | – | | |

A16 When do you tend to use an e-cigarette? (**Please tick all that apply**)

❑ When I get a craving to smoke or vape

❑ When I experience withdrawal symptoms like feeling restless, or irritable

❑ When I am feeling low

❑ When socialising

❑ When I see someone smoking

❑ First thing in the morning

❑ After a meal

❑ When I'm angry or stressed

❑ When I'm bored

❑ When I'm somewhere I can't smoke tobacco cigarettes

❑ When I don't want to or can't go outside to smoke (e.g. bad weather)

❑ Other. Please state: [ ]

❑ None of the above

A17 Please indicate how much you agree or disagree with each statement below.

(Select one answer per row)

	**Strongly disagree**	**Moderately disagree**	**Slightly disagree**	**Neither agree nor disagree**	**Slightly agree**	**Moderately agree**	**Strongly agree**
Using an e-cigarette is as satisfying as smoking	❑	❑	❑	❑	❑	❑	❑
Using an e-cigarette reduces / reduced my urge to smoke	❑	❑	❑	❑	❑	❑	❑
Using an e-cigarette has helped me to quit smoking	❑	❑	❑	❑	❑	❑	❑
Using an e-cigarette has helped me to reduce the number of tobacco cigarettes I smoke	❑	❑	❑	❑	❑	❑	❑
I would like to stop using e-cigarettes as well as tobacco cigarettes	❑	❑	❑	❑	❑	❑	❑

Participant ID number

		–				–			

FEEDING YOUR BABY

Everyone should complete this section (**Question B1 to B2**)

B1 Which statement best represents the way you plan to feed your new baby in the **first week** after birth? **(SELECT ONE OPTION)**

- ❑ I intend to breastfeed only (from the breast and/or expressed breast milk)
- ❑ I intend to formula feed only
- ❑ I intend to combine breastfeeding and formula feeding
- ❑ I am unsure

B2 Which statement best represents the way you plan to feed your new baby in the **first 6 months** after birth? **(SELECT ONE OPTION)**

- ❑ I intend to breastfeed only (from the breast and/or expressed breast milk)
- ❑ I intend to formula feed only
- ❑ I intend to combine breastfeeding and formula feeding
- ❑ I am unsure

YOUR SMOKING BEHAVIOUR AND BELIEFS

Everyone should answer this section (about tobacco smoking) (**Questions C1 to C4**)

C1 Please tick the box next to the box that best describes your smoking right now

- ❑ I don't smoke at all
- ❑ I smoke occasionally, but not every day
- ❑ I smoke every day, but have cut down during my pregnancy
- ❑ I smoke every day, about the same as before my pregnancy
- ❑ I smoke every day, and tend to smoke more than before my pregnancy

Participant ID number | | | – | | | – | | |

C2 Have you smoked at all since you completed the first questionnaire when you joined this study?

❑ Yes

❑ No

❑ I can't remember

❑ Not applicable – I have never smoked tobacco cigarettes

C3 Since you completed the first study questionnaire, have you tried to stop smoking?

❑ Yes

❑ No

❑ Not applicable - I haven't smoked at all since I completed the first questionnaire

❑ Not applicable - I have never smoked

C4 If you currently smoke, or have stopped smoking, please answer each of the following questions by circling the appropriate number. **Please circle one number per question.**

	**Not at all**	**A little**	**Moderately**	**Very much**	**Extremely**
How **determined** are you to stop smoking until your baby is born?	1	2	3	4	5
How **confident** are you that you can stop smoking until your baby is born?	1	2	3	4	5
How **determined** are you to stop smoking for good?	1	2	3	4	5
How **confident** are you that you can stop smoking for good?	1	2	3	4	5

If you **SMOKE EVERY NOW & AGAIN or MORE OFTEN THAN THIS** please answer **question**s **C5 to C7**

If you **DO NOT SMOKE AT THE MOMENT** you have finished – please return the questionnaire in the envelope provided. Thank you for your help.

Participant ID number | | | – | | | | – | | |

C5 Approximately how many cigarettes do you smoke each day?

- ❑ 0-5
- ❑ 6-10
- ❑ 11-15
- ❑16-20
- ❑ 21-30
- ❑ 31 or more

C6 How soon after you wake up do you smoke your first cigarette of the day?

- ❑ Within 5 minutes
- ❑ 6-30 minutes
- ❑ 31-60 minutes
- ❑ After 60 minutes

C7 Are you seriously planning to quit smoking?

- ❑ Yes, within the next 2 weeks
- ❑ Yes, within the next 30 days
- ❑ Yes, within the next 3 months
- ❑ No, I am not seriously planning to quit

Thank you for completing the questionnaire – we are very grateful for your help.

Please return in the envelope provided (no stamp required).
