# Supplementary material for: Smoking and vaping patterns during pregnancy and the postpartum: A longitudinal UK cohort survey
Source: Addict Behav. 2021 Dec;123:107050. doi: 10.1016/j.addbeh.2021.107050 (PMC8434421; doi:10.1016/j.addbeh.2021.107050)
Supplement: Supplementary data 4 [file mmc4.pdf]

University of Nottingham
UK | CHINA | MALAYSIA

Researchers' use only

**Participant ID number**			–				–			
	Site number				*Participant number*				*Initials*	
**Date returned**			/			/				
	D	*D*		*M*	*M*		*Y*	*Y*	*Y*	*Y*
**Researcher's initials**										

Pregnancy lifestyle survey 2017

Pregnant women's attitudes towards smoking and electronic cigarettes

FOLLOW UP 2 QUESTIONNAIRE
Final Version: 2.0
Version date: 30th October 2017

Please complete this questionnaire **within the next 2 weeks** and then return it in the envelope provided (no stamp required). Once you have completed and returned the questionnaire, we will send you a £10 shopping voucher to thank you for your time and support.

The information you give us will be anonymous and only used by the Pregnancy Lifestyle Survey researchers.

If you have any questions or concerns about this questionnaire, please call the Smoking and Pregnancy Research Office on 0115 XXX XXXX

Thank you for your help

Today's date (please enter the date you completed this):

………… /…………… /…………

dd / mm / yyyy

Participant ID number | | | – | | | – | | |

YOUR EXPERIENCE OF E-CIGARETTES

In this section, **we would like you to answer some questions about your experiences of and views on e-cigarettes – even if you haven't used them.**

Some questions are for everyone and others are for particular individuals (e.g., those currently using e-cigarettes).

Everyone should answer A1 to A8 below. After that, please follow the instructions about which questions to answer. Thank you.

A1 How often did you use an e-cigarette or vaping device during each of the following times? (Please tick one box for each time period)

	Not used at all	Only used once or twice	Used occasionally, but less than weekly	Used less than daily, but at least once a week	Used every day
In the week before the birth of your baby	❑	❑	❑	❑	❑
In the first 2 months after the birth of your baby	❑	❑	❑	❑	❑
Now	❑	❑	❑	❑	❑

A2 Now that you've had your baby, does anyone you know think you should use an e-cigarette instead of smoking cigarettes? (tick as many as apply)

❑Yes, my partner

❑ Yes, another family member or friend

❑ Yes, a health professional (e.g., health visitor, midwife, stop smoking advisor, GP)

❑Yes, someone else I know

❑ No

❑ I have not discussed this with anyone

Participant ID number | | | – | | | | – | | |

A3 We would like to know your views on using e-cigarettes after having your baby.

Please indicate how much you agree or disagree with each statement below.

(Select one answer per row)

	**Strongly disagree**	**Moderately disagree**	**Slightly disagree**	**Neither agree nor disagree**	**Slightly agree**	**Moderately agree**	**Strongly agree**
E-cigarettes should be promoted to help women who have recently given birth to ***stop*** smoking	❑	❑	❑	❑	❑	❑	❑
E-cigarettes should be promoted to help women who have recently given birth to help prevent them from ***restarting*** smoking	❑	❑	❑	❑	❑	❑	❑
E-cigarettes should be promoted to help people with babies and young children keep their homes smoke free	❑	❑	❑	❑	❑	❑	❑
Now that I have had my baby, I'd be more likely to use an e-cigarette if a health professional recommended it	❑	❑	❑	❑	❑	❑	❑
Now I have had my baby I would feel more comfortable using an e-cigarette in public compared to when I was pregnant	❑	❑	❑	❑	❑	❑	❑
E-cigarettes should only be used in the home or private places	❑	❑	❑	❑	❑	❑	❑
I would be happy for e-cigarettes to be used in my home when my baby is in the ***same*** room	❑	❑	❑	❑	❑	❑	❑
I would be happy for e-cigarettes to be used in my home when my baby is in a ***different*** room	❑	❑	❑	❑	❑	❑	❑
I would be concerned that if my child saw me using e-cigarettes it would be setting them a bad example	❑	❑	❑	❑	❑	❑	❑

Participant ID number | | | – | | | | – | | |

A4 We would like to know your views on the safety of e-cigarettes ***after having a baby***.

Please indicate how much you agree or disagree with each statement below.

(Select one answer per row)

	**Strongly disagree**	**Moderately disagree**	**Slightly disagree**	**Neither agree nor disagree**	**Slightly agree**	**Moderately agree**	**Strongly agree**
Using e-cigarettes around my baby may cause them harm	❑	❑	❑	❑	❑	❑	❑
Using e-cigarettes around my baby is much less harmful than smoking tobacco cigarettes	❑	❑	❑	❑	❑	❑	❑
E-cigarettes should only be available on prescription	❑	❑	❑	❑	❑	❑	❑

Participant ID number | | | – | | | | – | | |

A5 Below are some reasons people may give for using e-cigarettes ***after having a baby***.

In the first column, please tick the reason that you think is the ***most important***. In the second column, tick any other reasons that you think are important for using e-cigarettes after having a baby.

	Most important reason for using e-cigarettes **(tick one only)**	Other reasons for using e-cigarettes **(tick any you think are important)**
To help me quit smoking	❑	❑
To help me cut down the number of tobacco cigarettes	❑	❑
They are healthier than tobacco cigarettes	❑	❑
They are cheaper than tobacco cigarettes	❑	❑
They are less harmful to others around me than tobacco cigarettes	❑	❑
They feel similar to smoking	❑	❑
They look fashionable / stylish	❑	❑
They taste and smell nicer than tobacco cigarettes	❑	❑
To avoid my home or clothes smelling of tobacco smoke	❑	❑
I can use them in places where smoking is banned	❑	❑
It is more acceptable to be seen using e-cigarettes than smoking tobacco cigarettes	❑	❑
Other reason (please state) ………………………………………………………………………………	❑	❑

Participant ID number | | | – | | | – | | |

A6 Below are some reasons people may give for **NOT** using e-cigarettes ***after having a baby***.

In the first column, please tick the reason that you think is the ***most important***. In the second column, tick any other reasons that you think are important for not using e-cigarettes after having a baby.

	Most important reason for NOT using e-cigarettes **(tick one only)**	Other important reasons for NOT using e-cigarettes **(tick any you think are important)**
Bad stories in the press or on social media put me off them	❑	❑
Family or friends tell me not to use them	❑	❑
They don't get rid of the 'smoking habit'	❑	❑
They don't get rid of nicotine addiction	❑	❑
They don't satisfy my cravings for tobacco cigarettes	❑	❑
There isn't enough research on them	❑	❑
I'd be too embarrassed to use them	❑	❑
I'd rather try other ways to quit smoking, such as nicotine patches	❑	❑
I'm worried about side effects	❑	❑
I get different messages from different people so I'm confused	❑	❑
Other reason (please state) …………………………………………………………………………………………	❑	❑

Participant ID number | | | – | | | | – | | |

A7 How likely are you to try or use e-cigarettes in the future?

❑ Very likely

❑ Somewhat likely

❑ Neither likely nor unlikely (neutral)

❑ Somewhat unlikely

❑ Very unlikely

❑ I don't know

A8 We would like to know about smoking and e-cigarette use in your home.

(**Please select one answer for each question)**

	Never	Almost never	Sometimes	Fairly often	Very often
How often do you smoke tobacco cigarettes in your home nowadays?	❑	❑	❑	❑	❑
How often do other people smoke tobacco cigarettes in your home nowadays?	❑	❑	❑	❑	❑
How often do you use an e-cigarette in your home nowadays?	❑	❑	❑	❑	❑
How often do other people use an e-cigarette in your home nowadays?	❑	❑	❑	❑	❑

If you **currently use e-cigarettes, even if this is not every day**, please answer **Questions A9 to A17**

If you **do not currently use e-cigarettes**, please **go to Question B1 on page 11**

A9 How soon after you wake up do you first use your e-cigarette?

❑ Within 5 minutes

❑ 6-30 minutes

❑ 31-60 minutes

❑ After 60 minutes

Participant ID number | | | – | | | | – | | |

A10 What type of electronic cigarette or vaping device do you use the most?
SELECT ONE OPTION

❑ A disposable e-cigarette or vaping device (non–rechargeable)

❑ A rechargeable e-cigarette or vaping device that uses replaceable pre-filled cartridges

❑ A rechargeable e-cigarette or vaping device with a tank that you can refill with fluids

❑ A rechargeable modular system that you refill with liquids (you use your own combination of separate parts: battery, atomiser, fluid etc.)

❑ Don't know

A11 What strength of e-liquid / fluid / juice / cartridge do you usually use?
SELECT ONE OPTION

❑ Zero (contains no nicotine)

❑ Low (up to 6mg (0.6%) nicotine)

❑ Medium (between 7 and 12mg (0.7-1.2%) of nicotine)

❑ High (between 13 and 20mg (1.3-2.0%) of nicotine)

❑ Very high (more than 20mg (2.0%) of nicotine)

❑ It contains nicotine, but I don't know what strength

❑ I don't know if it contains nicotine

A12 Please try to estimate how much on average you use your e-cigarette per day

Number of mls of fluid / juice	
Number of cartridges	
Don't know	❑

A13 Have you changed how much you use e-cigarettes **since giving birth**?
SELECT ONE OPTION

❑ Yes, I tend to use them **less** now

❑ Yes, I tend to use them **more** now

❑ No, I tend to use them about the **same** now

❑ Not applicable. I didn't using an e-cigarette during my pregnancy

❑ Don't know / can't remember

A14 Which is your preferred e-cigarette flavour category?
SELECT ONE OPTION

❑ Tobacco

❑ Tobacco menthol, menthol or mint

❑ Some other flavour like fruit, candy, alcohol, coffee, vanilla etc.

❑ No flavour

❑ Don't know

A15 What is your main reason for using an e-cigarette? (**SELECT ONE OPTION**)

❑ To quit smoking

❑ To cut down smoking

❑ To use when I cannot or am not allowed to smoke

❑ To avoid returning to smoking

❑ Because I enjoy it

❑ Curiosity / just wanted to try them

❑ To use instead of smoking tobacco cigarettes around my baby

❑ Some other reason (please state) [ ]

❑ Don't know

Participant ID number

		–				–			

A16 When are you likely to use an e-cigarette? (**Please tick all that apply**)

- ❑ When I get a craving to smoke or vape
- ❑ When I experience withdrawal symptoms like feeling restless, or irritable
- ❑ When I am feeling low
- ❑ When socialising
- ❑ When I see someone smoking
- ❑ First thing in the morning
- ❑ After a meal
- ❑ When I'm angry or stressed
- ❑ When I'm bored
- ❑ When I'm somewhere I can't smoke tobacco cigarettes
- ❑ When I don't want to or can't go outside to smoke (e.g. bad weather, or can't leave the baby)
- ❑ Other. Please state: ____________________
- ❑ None of the above

Participant ID number | | | – | | | | – | | |

A17 Please indicate how much you agree or disagree with each statement below.

(Select one answer per row)

	**Strongly disagree**	**Moderately disagree**	**Slightly disagree**	**Neither agree nor disagree**	**Slightly agree**	**Moderately agree**	**Strongly agree**
Using an e-cigarette is as satisfying as smoking	❑	❑	❑	❑	❑	❑	❑
Using an e-cigarette reduces / reduced my urge to smoke	❑	❑	❑	❑	❑	❑	❑
Using an e-cigarette has helped me to quit smoking	❑	❑	❑	❑	❑	❑	❑
Using an e-cigarette has helped me to reduce the number of tobacco cigarettes I smoke	❑	❑	❑	❑	❑	❑	❑
I don't want to stop smoking completely. I want to carry on smoking tobacco cigarettes as well as using e-cigarettes	❑	❑	❑	❑	❑	❑	❑
I would like to stop using e-cigarettes as well as tobacco cigarettes	❑	❑	❑	❑	❑	❑	❑

Participant ID number [ ][ ] – [ ][ ][ ] – [ ][ ][ ]

YOUR BABY

Everyone should complete this section (**Questions B1 to B7**)

B1 Is your baby one of twins, triplets or other multiple birth?

- ❑ No
- ❑ Yes, twin
- ❑ Yes, triplets or other multiple birth

If you have twins or triplets, please complete the questions in this section about the baby who was born first.

B2 How much did your baby weigh when he/she was born?

Either in pounds and ounces:

[ ] [ ] lb [ ] [ ] oz

Or in kilograms:

[ ] • [ ] [ ] kg

- ❑ Don't know

B3 Which statement best represents the way you planned to feed your new baby **in the first week after birth**? **(SELECT ONE OPTION)**

- ❑ I intended to breastfeed only (from the breast and/or expressed breast milk)
- ❑ I intended to formula feed only
- ❑ I intended to combine breastfeeding and formula feeding
- ❑ I was unsure

Participant ID number | | | – | | | | – | | | |

B4 Which statement best represents the way you fed your new baby **in the first week after birth**? **(SELECT ONE OPTION)**

❑ I breastfed exclusively from birth (from the breast and/or expressed breast milk)

❑ I formula fed exclusively from birth

❑ I combined breastfeeding and formula feeding from birth

B5 Which statement best represents the way you feed your baby **now**? **(SELECT ONE OPTION)**

❑ I am just breastfeeding (from the breast and/or expressed breast milk)

❑ I am just formula feeding

❑ I combine breastfeeding and formula feeding

B6 How old was your baby when they last had breast milk (from the breast or expressed breast milk)? **(SELECT ONE OPTION)**

❑ Never had breast milk

❑ Less than 1 week

❑ Less than 1 month

❑ Over 1 month, but I've stopped breastfeeding now

❑ I'm still breastfeeding

B7 We would like to know your views on e-cigarettes, smoking and breastfeeding.

Please indicate how much you agree or disagree with each statement below.

(Select one answer per row)

	**Strongly disagree**	**Moderately disagree**	**Slightly disagree**	**Neither agree nor disagree**	**Slightly agree**	**Moderately agree**	**Strongly agree**
Mothers should not breastfeed if they smoke tobacco cigarettes	❑	❑	❑	❑	❑	❑	❑
Mothers should not breastfeed if they use e-cigarettes	❑	❑	❑	❑	❑	❑	❑
Using e-cigarettes when breastfeeding is safe for the baby	❑	❑	❑	❑	❑	❑	❑
Using e-cigarettes when breastfeeding is as safe for the baby as using nicotine patches	❑	❑	❑	❑	❑	❑	❑
If a mother breastfeeds, it is safer for the baby if she uses an e-cigarette than if she smokes tobacco cigarettes	❑	❑	❑	❑	❑	❑	❑

Participant ID number | | | – | | | | – | | |

YOUR SMOKING BEHAVIOUR AND BELIEFS

Everyone should answer this section (about tobacco smoking) (**Questions C1 to C5**)

C1 Please tick the box next to the statement that best describes your smoking right now

❑ I don't smoke at all

❑ I smoke occasionally, but not every day

❑ I smoke every day, but less than when I was pregnant

❑ I smoke every day, about the same as when I was pregnant

❑ I smoke every day, and tend to smoke more than when I was pregnant

C2 Did you smoke at all in the week before the birth of your baby?

❑ Yes ❑ No

❑ I can't remember ❑ Not applicable - I have never smoked

C3 Have you smoked at all since the birth of your baby?

❑ Yes ❑ No

If Yes, how soon after the birth of your baby did you first smoke?

❑ Within 24 hours ❑ 1-2 months

❑ 1-6 days ❑ More than 2 months

❑ 7-30 days

C4 Since the birth of your baby, have you tried to stop smoking?

❑ Yes ❑ No

❑ Not applicable - I haven't smoked since the birth of my baby ❑ Not applicable - I have never smoked

Participant ID number | | | – | | | – | | |

C5 If you currently smoke, or have stopped smoking, please answer each of the following questions by circling the appropriate number. **Please circle one number per question.**

	**Not at all**	**A little**	**Moderately**	**Very much**	**Extremely**
How **determined** are you to stop smoking for good?	1	2	3	4	5
How **confident** are you that you can stop smoking for good?	1	2	3	4	5

C6 **Before your last pregnancy** had you **ever** tried any of the following to help you stop smoking? (**Please tick all that apply**)

- ❑ Talked to your GP or a nurse about giving up smoking
- ❑ Talked to a midwife about giving up smoking
- ❑ Attended a NHS stop smoking service group session
- ❑ Attended a solo/individual NHS stop smoking service session (i.e. not with other people)
- ❑ Called a stop smoking telephone helpline
- ❑ Used Nicotine Replacement Therapy (NRT) (e.g. nicotine patches or gum)
- ❑ Set a quit date
- ❑ Other. Please state:
- ❑ None of the above

C7 **When you were pregnant**, did you try any of the following to help you stop smoking / remain stopped? (**Please tick all that apply**)

- ❑ Talked to your GP or a nurse about giving up smoking
- ❑ Talked to a midwife about giving up smoking
- ❑ Attended a NHS stop smoking service group session
- ❑ Attended a solo/individual NHS stop smoking service session (i.e. not with other people)
- ❑ Called a stop smoking telephone helpline
- ❑ Used Nicotine Replacement Therapy (NRT) (e.g. nicotine patches or gum)
- ❑ Set a quit date
- ❑ Other. Please state:
- ❑ None of the above

Participant ID number

		–				–			

C8 **Since having your baby**, have you tried any of the following to help you stop smoking / remain stopped? (**Please tick all that apply**)

- ❑ Talked to your GP or a nurse about giving up smoking
- ❑ Talked to a midwife or health visitor about giving up smoking
- ❑ Attended a NHS stop smoking service group session
- ❑ Attended a solo/individual NHS stop smoking service session (i.e. not with other people)
- ❑ Called a stop smoking telephone helpline
- ❑ Used Nicotine Replacement Therapy (NRT) (e.g. nicotine patches or gum)
- ❑ Set a quit date
- ❑ Other. Please state:
- ❑ None of the above

If you **SMOKE EVERY NOW & AGAIN or MORE OFTEN THAN THIS** please answer **questions C6 to C8**

If you **DO NOT SMOKE AT THE MOMENT** you have finished – please return the questionnaire in the envelope provided. Thank you for your help.

C9 Approximately how many cigarettes do you smoke each day?

❑ 0-5	❑ 16-20
❑ 6-10	❑ 21-30
❑ 11-15	❑ 31 or more

C10 How soon after you wake up do you smoke your first cigarette of the day?

❑ Within 5 minutes	❑ 31-60 minutes
❑ 6-30 minutes	❑ After 60 minutes

Participant ID number

		–				–			

C11 Are you seriously planning to quit smoking?

- ❑ Yes, within the next 2 weeks
- ❑ Yes, within the next 30 days
- ❑ Yes, within the next 3 months
- ❑ No, I am not seriously planning to quit

Thank you for completing the questionnaire – we are very grateful for your help.

Please return in the envelope provided (no stamp required).
